# Peculiarities of One-Carbon Metabolism in the Strict Carnivorous Cat and the Role in Feline Hepatic Lipidosis

**DOI:** 10.3390/nu5072811

**Published:** 2013-07-19

**Authors:** Adronie Verbrugghe, Marica Bakovic

**Affiliations:** 1 Department of Clinical Studies, Ontario Veterinary College, University of Guelph, 50 Stone Road East, Guelph, ON N1G 2W1, Canada; 2 Department of Human Health and Nutritional Sciences, College of Biological Science, University of Guelph, Guelph, ON N1G 2W1, Canada; E-Mail: mbakovic@uoguelph.ca

**Keywords:** B-vitamins, carnivore, essential amino acids, essential fatty acids, fatty liver, feline, metabolism, methyl donor, nutrition, protein

## Abstract

Research in various species has indicated that diets deficient in labile methyl groups (methionine, choline, betaine, folate) produce fatty liver and links to steatosis and metabolic syndrome, but also provides evidence of the importance of labile methyl group balance to maintain normal liver function. Cats, being obligate carnivores, rely on nutrients in animal tissues and have, due to evolutionary pressure, developed several physiological and metabolic adaptations, including a number of peculiarities in protein and fat metabolism. This has led to specific and unique nutritional requirements. Adult cats require more dietary protein than omnivorous species, maintain a consistently high rate of protein oxidation and gluconeogenesis and are unable to adapt to reduced protein intake. Furthermore, cats have a higher requirement for essential amino acids and essential fatty acids. Hastened use coupled with an inability to conserve certain amino acids, including methionine, cysteine, taurine and arginine, necessitates a higher dietary intake for cats compared to most other species. Cats also seemingly require higher amounts of several B-vitamins compared to other species and are predisposed to depletion during prolonged inappetance. This carnivorous uniqueness makes cats more susceptible to hepatic lipidosis.

## 1. Introduction

Non-alcoholic fatty liver disease (NAFLD), the hepatic manifestation of metabolic syndrome, consists of a spectrum ranging from simple triacylglycerol (TAG) accumulation in the hepatocytes (hepatic steatosis) to steatosis with inflammation (steatohepatitis), fibrosis and cirrhosis [1]. Human and animal studies have proposed pathophysiological mechanisms for the progression of NAFLD from steatosis to steatohepatitis. These findings suggest that hepatic steatosis is related to excessive delivery of fatty acids to the liver caused by increased whole body rate of lipolysis, due to systemic insulin resistance, coupled with increased hepatic *de novo* lipogenesis and attenuated export of hepatic TAG [2]. In addition, due to a high rate of fatty acid oxidation in the liver, there is increased oxidative stress, leading to changes in mitochondrial function, depletion of ATP, DNA damage, lipid peroxidation, release of cytokines and, consequently, hepatic inflammation and fibrosis [3]. The increase in oxidative stress results in augmented consumption of the major intracellular antioxidant, glutathione (GSH). In addition, as a result of high fatty acid uptake by the liver coupled with higher *de novo* lipogenesis, a higher rate of carnitine and phosphatidylcholine synthesis is required to enable fatty acid oxidation and export of very-low-density lipoproteins (VLDL), respectively.

The majority of research into the etiology and pathophysiology of NAFLD and progression to hepatic steatosis has been performed using rodent models. Although these models have provided vital insights into the pathogenesis of steatosis and steatohepatitis, these models are often disappointing, especially as no existing model exhibits the entire NAFLD phenotype as encountered in clinical practice, and many differ from the human disease in all but gross histological appearance [4,5,6]. The lack of a reliable model has hampered research in this field. Therefore, additional comprehensive animal models are warranted and should have a liver pathology that features steatosis, inflammation, liver cell injury, including ballooning hepatocyte degeneration in addition to simply fatty change, and fibrosis. Additionally, the animal model must exhibit metabolic abnormalities, such as obesity, insulin resistance, hyperglycaemia, dyslipidemia and altered adipokine profile [6].

The domestic cat has previously been shown to be an appropriate model for examining human metabolic diseases, particularly diabetes mellitus [7,8]. Similarities between feline and human diabetes include insulin resistance, hyperglycaemia, pancreatic islet cell lesions and partial loss of pancreatic β-cells. Furthermore, as with humans, obesity, which is also becoming increasingly prevalent in cats [9], is a risk factor for feline diabetes [7,8,9,10] and feline hepatic steatosis, also called feline hepatic lipidosis (FHL) [11,12,13,14]. Still, despite these similarities, a thorough understanding of the peculiarities of the feline protein, one-carbon and fatty acid metabolism and their involvement in the pathophysiology of FHL is desirable if the feline model is to be pursued as a viable alternative to the use of rodents in this perspective and will be the focus of this review.

## 2. Feline-Specific Metabolic Features

Cats are obligate carnivores. From a nutritional perspective, this means that in their natural habitat, cats consume prey and rely on nutrients in animal tissues. Due to evolutionary pressure, cats have developed several physiological and metabolic adaptations, including a number of peculiarities in protein, one-carbon and fatty acid metabolism that have led to specific and unique nutritional requirements [15,16,17,18,19].

**Table 1:** Dietary requirements for protein, selected amino acids and B-vitamins linked to one-carbon metabolism in cats, dogs, mink, laboratory rat and male and female human beings.

Amount/kg BW^0.75^	Cat ^(1)^	Mink ^(2)^	Dog ^(3)^	Rat ^(4)^	Human Being ^(5)^	
					Male	Female
Protein (g)	4.48	7.81	3.28	1.47	2.31	1.90
Arginine (g)	0.17	NA	0.11	0.13 †	NE	NE
Methionine (g)	0.038	NA	0.11	NA	NA	NA
Methionine + Cysteine (g)	0.076	NA	0.21	0.068	0.055	0.055
Taurine (g)	0.009	NA	NE	NE	NE	NE
Cobalamin (μg)	0.50	0.98 *	1.15	1.47 †	0.099	0.099
Choline (mg)	57.0	NA	56.0	22.1 †	22.7	17.6
Folate (μg)	16.8	15.0 *	8.9	29. 47 †	16.5	16.5
Pyridoxine (mg)	0.056	0.048 *	0.049	0. 177 †	0.054	0.054

BW: body weight; NA: not available; NE: non-essential. ^(1)^ Based on recommended allowances for adult cats expressed as amount per 100 kcal, calculated for a 4 kg cat with a maintenance energy intake of 100 kcal × BW^0.67^ [20]. ^(2)^ Based on estimated nutrient requirements expressed as amount per kg diet, calculated for a 600 g mink with a maintenance energy intake of 140 kcal × BW and a diet containing 4.1 kcal/g [21]. * For B-vitamins, requirements are presented for growth, as separate requirements for maintenance have not been determined. ^(3)^ Recommended allowances for adult dogs expressed as amount per kg BW^0.75^ [20]. ^(4)^ Based on estimated nutrient requirements expressed as amount per kg diet, calculated for a 300 g rat with a maintenance energy intake of 112 kcal × BW^0.75^ and a diet containing 4.08 kcal/g [22]. † For arginine and B-vitamins, requirements are presented for growth, as separate requirements for maintenance have not been determined. ^(5)^ Based on dietary reference intakes for men and women from 31 to 50 years of age expressed as amount per day, calculated for a 70 kg person [23,24].

### 2.1. Dietary Protein Requirement

Cats have higher dietary protein requirements compared to omnivores and herbivores (Table 1), high endogenous nitrogen losses, high *in vitro* activities of enzymes involved in protein catabolism and appear to have limited ability to adjust protein oxidation to low dietary intakes of protein [19]. In the past, this has been attributed to a lack of metabolic flexibility. Omnivorous and herbivorous species have the capacity to adapt to various levels of protein intake [25,26,27,28]. When an animal is fed a high protein diet, the activities of the amino acid catabolic enzymes in the liver and kidney are increased, facilitating disposal of excess nitrogen. The opposite is true; if a lower than normal level of protein is fed, the amino acid catabolism decreases, enabling the animal to preserve amino acids. Still, in cats, it was found that the activities of none of the hepatic catabolic enzymes changed when cats were shifted from a high protein diet (70% soy protein) to a low protein diet (17% soy protein) and *vice versa* [29]. It was therefore thought that cats have only limited capabilities for enzyme adaptation as compared to herbivores and omnivores and that the hepatic enzymes that catabolise amino acids are set at a permanently high level. This may indicate that the cat is wasteful of amino acids, cannot conserve nitrogen and, therefore, has a high obligatory nitrogen loss. Still, *in vitro* adaptation of protein oxidation to dietary protein content (17.5% *versus* 70% soy protein) was demonstrated by Silva and Mercer [30]. Furthermore, also, Russell *et al*. concluded, based on ureakinetics [31] and indirect calorimetry [32], that the absolute amount of protein catabolised does vary with dietary protein intake (35% of metabolisable energy (ME) *versus* 52% ME). Green *et al.* concluded, based on indirect calorimetry in cats fed four different levels of dietary protein (7.5% ME, 14.2% ME, 27.1% ME and 49.6% ME), that cats can indeed adapt to a wide range of dietary protein intake (14%–50% ME), provided the minimum protein requirement for maintenance (10%–11% ME) [20] is met [33]. When cats were fed a diet that contained sufficient protein to meet their maintenance requirement, protein intake and protein oxidation were closely matched, and nitrogen balance was maintained. However, cats were unable to fully adapt; protein oxidation exceeded protein intake and the nitrogen balance was negative when dietary protein intake was below the maintenance protein requirement [33].

Why cats cannot adjust the catabolism of amino acids to lower intakes of protein sufficient for other species is not clear, especially as the overall rate of protein turnover in cats is at the lower end of the range for omnivores and herbivores [34]. Recently, Eisert proposed a mechanistic explanation for this paradox [19]. As cats have a relatively large brain, a significant proportion of protein must be diverted into gluconeogenesis to supply the brain. The high protein requirement in felines is therefore the result of a high metabolic demand for glucose that must be met by amino acid-based gluconeogenesis [19]. Indeed, higher activities of rate limiting enzymes of gluconeogenesis, *i.e.*, pyruvate carboxylase, fructose-1,6-biphosphatase and glucose-6-phosphatase, were observed in feline livers compared to in canine livers [35,36]. Furthermore, feeding a low protein diet did not downregulate the hepatic gluconeogenic enzyme activities [29]. Furthermore, in cats, the obligatory amino acid oxidation in the fed state is considerably higher than the fasting amino acid oxidation [19], which is in accordance with the fact that the maximal gluconeogenic capacity and activities of gluconeogenic enzymes in the fed state are as high as, or even higher than, during food deprivation [29,37]. Therefore, cats do not have a high protein requirement *per se*, but rather a secondarily elevated protein requirement in response to a high endogenous glucose demand. Still, during starvation the high rate of amino acid catabolism becomes a disadvantage and puts cats at risk for protein malnutrition and essential amino acid deficiency.

### 2.2. One-Carbon Metabolism

The first step in mammalian methionine metabolism is conversion of methionine and ATP by l-methionine *S*-adenosyltransferase (MAT) to *S*-adenosylmethionine (SAMe), a universal methyl donor for all methylation reactions [38,39]. The methyl group of SAMe is transferred to a large variety of methyl acceptors, including nucleic acids, proteins, lipids and secondary metabolites, with formation of *S*-adenosylhomocysteine (SAH). For example, methylation of glycine to sarcosine by glycine *N*-methyltransferase (GNMT), methylation of guanidinoacetate by guanidinoacetate *N*-methyltransferase (GAMT) to form creatine, methylation of phosphatidylethanolamine (PE) to phosphatidylcholine by PE *N*-methyltransferase (PEMT) and methylation of lysine by lysine *N*-methyltransferase to form 6-*N*-trimethyllysine, which is the main precursor for l-carnitine synthesis [40,41,42]. Once the methyl group is transferred to a substrate by the appropriate methyltransferase, the SAH is rapidly hydrolysed to homocysteine and adenosine by SAH hydrolase [38,39]. Homocysteine may be remethylated to regenerate methionine using folate (vitamin B_9_). Through methionine synthase, a methyl group is transferred from 5-methyltetrahydrofolate (5-CH_3_-THF) to cobalamin (vitamin B_12_) to form methylcobalamin. The methylcobalamin eventually transfers the methyl group from homocysteine to produce methionine [38]. An alternative folate-independent pathway utilises betaine, derived from oxidation of choline, as a methyl donor in a reaction catalysed by betaine-homocysteine methyltransferase to produce methionine and dimethylglycine [38,39]. Furthermore, homocysteine can also be catabolised via the transsulfuration pathway through the action of the pyridoxal phosphate (vitamin B_6_)-containing enzymes, cystathionine-β-synthase and cystathionase, leading to production of cysteine and its derivatives, GSH, taurine and inorganic sulphur [38,39]. The carbon skeleton of methionine, α-ketobutyrate, is eventually oxidatively decarboxylated to propionyl-CoA, which enters the citric acid cycle at the level of succinyl-CoA and can be used for gluconeogenesis [38].

Table 1 compares the dietary requirements for amino acids (methionine, cysteine, taurine and arginine) and B-vitamins (cobalamin, choline, folate and pyridoxine) linked to one-carbon metabolism in cats, minks, dogs, laboratory rats and human beings.

#### 2.2.1. Essential Amino Acids

**Figure 1:**
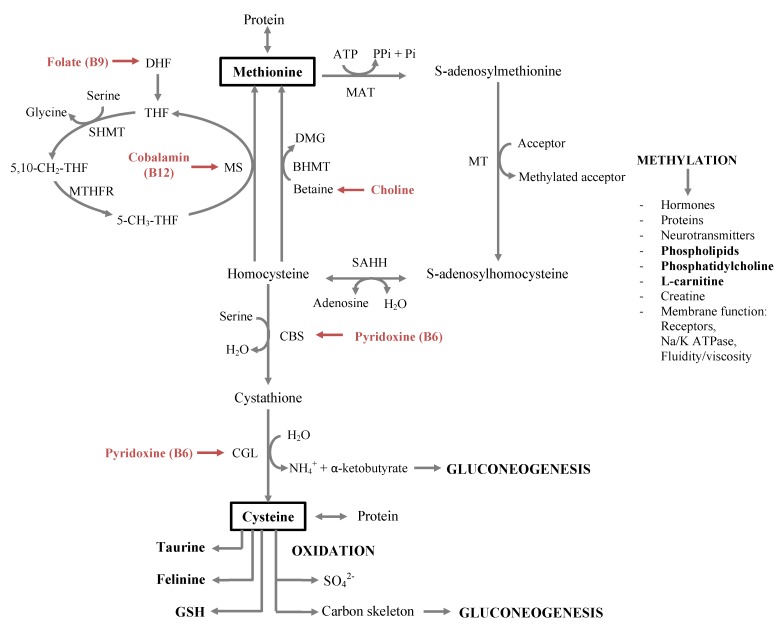
Major pathways of sulphur-containing amino acid metabolism in the feline, involving the esstential amino acids, methionine, cysteine and taurine, and the B-vitamines, cobalamin, choline, folate and pyridoxine.5-CH_3_-THF: 5-methyl-tetrahydrofolate; 5,10-CH_2_-THF: 5,10-methylene-tetrahydrofolate; BHMT: betaine-homocysteine methyltransferase; CBS: cystathione β-synthase; CGL: cystathione γ-lyase; DMG: dimethyl glycine; MAT: methionine adenosyltransferase; MS: methionine synthase; MT: various methyltransferases; MTHFR: methylenetetrahydrofolate reductase; Pi: orthophosphate; Pii: pyrophosphate; SAHH: *S*-adenosylhomocysteine hydrolyase; SHMT: serine hodroxymethyltransferase; THF: tetrahydrolfolate.

Cats have an increased need for specific essential amino acids. As other animals, cats have a dietary need for the following nine amino acids: histidine, isoleucine, leucine, lysine, methionine, phenylalanine, threonine, tryptophan and valine. Yet, higher requirements are reported for sulfur-containing amino acids, methionine, cysteine and taurine, as well as for arginine (Table 1) [15,16,17]. The major pathways of sulphur-containing amino acid metabolism in the feline are summarised in Figure 1.

Methionine is generally the most limiting amino acid in a diet formulated for cats using ingredients from animal sources and is part of the coenzyme, SAMe, an important methyl donor essential for methylation reactions. More than 60 metabolic reactions involve the transfer of a methyl group from SAMe to various substrates, playing an important role in a multitude of metabolic pathways, such as cell replication, synthesis of neurotransmitters, phosphatidylcholine, phospholipids and l-carnitine, membrane function, *etc*. [39]. Moreover, during catabolism, the carbon skeleton of methionine is gluconeogenic and cysteine can be synthetized in the body from the sulfur atom from methionine and the dispensable amino acid, serine [39]. As in other species, cysteine is a dispensable amino acid that, provided adequate methionine is available, can contribute significantly to the total sulfur-containing amino acid content by the irreversible conversion of methionine to cysteine. There is not an inordinate quantity of methionine converted to cysteine; about half of the methionine requirement is spared by cysteine [43]. Cysteine is an important component of proteins for their secondary structure [39] and is a major constituent of hair [44]. Cysteine also functions as an essential thiol donor for hepatocellular glutathione synthesis [39] and as a precursor for felinine [45,46] and taurine [39].

Felinine is a unique branched-chain sulfur-containing amino acid detected in the urine of only certain members of the Felidae family, including domestic cats [47]. It shows structure similarity with isovalthine, which is found in cat urine, but also in hypercholesterolemic human beings [48]. The carbon backbone of the side chain of both felinine and isovalthine appears to be derived from isoprenoid units similar to that used to synthesize cholesterol [47]. Consequently, both have been hypothesized to be involved in the regulation of cholesterol metabolism [47]. However, the fact that felinine excretion is gender specific [49,50] supports the suggested biological role as a pheromone involved in territorial marking [15,47].

The cat is also unique in its need for dietary taurine, a β-amino-sulphonic acid, not a protein component, but the most abundant free amino acid in animal tissues. Decreased activity and concentration of cysteinesulfinic acid decarboxylase limits taurine synthesis in cats [51]. Furthermore, some of the cysteine is shunted toward synthesis of felinine [47]. Cats also have a markedly higher physiological demand for taurine, characterised by an obligate loss of taurine in bile. In contrast to other animals, cats do not use glycine, but rely almost exclusively on taurine to conjugate bile acids into bile salts, regardless of the dietary taurine level [52,53]. Furthermore, free taurine is wasted substantially, as deconjugation of taurine-conjugated bile salt and enterohepatic recovery is limited [54]. Furthermore, extensive bacterial degradation of taurine was observed in cats [55,56]. In addition to its importance in normal bile salt function and cholesterol excretion, taurine has many fundamental physiological roles, such as osmoregulation, ion transport, membrane stabilization, antioxidation, host-defense, inhibition of nerve pulses, adipose tissue regulation and fetal development [57,58,59,60,61]. In cats, aside from the specific pathological conditions, including feline central retina degeneration [62,63], reproductive failure in queens with associated congenital abnormalities in kittens [64,65] and dilated cardiomyopathy [66], taurine deficiency may also cause fat accumulation in the liver [67].

It has also been suggested that the cat’s higher requirement for sulphur-containing amino acids is related to the cat’s thick hair [15]. However, another more plausible explanation involves the fat content of the feline diet. As in their natural habitat, cats eat a high fat diet; perhaps an increased need for phospholipids for absorption and transport of fat has resulted in a higher demand for SAMe, necessary for methylation reactions and phospholipid synthesis [15]. A high activity of MAT would also result in an increased need for SAMe for methylation reactions [15]; yet, cats were found to increase the activity of MAT with increased dietary methionine levels, but the activity remained lower than that previously found in rats [68].

A last explanation could be found in the fact that the carbon skeleton of methionine is gluconeogenic and the cat’s preferential use of protein as an energy source and a priority of gluconeogenesis for amino acids, e.g., methionine [15,16]. Indeed, Eisert proposed that cats, being a small mammal with a large brain, have a high brain glucose requirement that needs to be met by *de novo* production of glucose from amino acids, which could explain the elevated protein and amino acid requirement of cats [19].

Most probably there is not one single reason that provides an explanation why cats have much higher dietary needs for sulfur-containing amino acids, but more likely, the reason is multifactorial, and all of the aspects of the feline sulfur-containing amino acid metabolism mentioned above play a smaller or a bigger role in the cat’s unique dietary requirements.

At last, a dietary source of arginine, also a gluconeogenic amino acid and an intermediate of the urea cycle, is required in both cats [69,70] and dogs [71], but the consequences of arginine deprivation are extremely dramatic in cats. Even a single arginine-free meal fed to cats may cause severe hyperammonemia two to five hours later [69]. Cats will die if fed an arginine-free diet, while dogs merely show signs of unthriftness and will occasionally vomit. The susceptibility of felids to arginine-deficiency hyperammonemia is related to their inability to synthetize ornithine in the intestine from glutamine and glutamic acid. Two feline mucosal enzymes involved in *de novo* ornithine synthesis are very low in activity: proline-5-carboxylase synthase [72] and ornithine aminotransferase [73]. Ornithine addition to arginine-free diets of cats completely prevented hyperammoniemia, however, without effect on weight loss [74]. Unlike ornithine, dietary citrulline can replace arginine and allow growth in young animals [74]. Aside from its urea cycle function, arginine may also affect liver lipid metabolism as a component of apolipoprotein E [75].

#### 2.2.2. B-Vitamins Involved in One-Carbon Metabolism

Cats seemingly require higher amounts of several B-vitamins compared to other species (Table 1) and are therefore predisposed to depletion during prolonged inappetance, maldigestion and malassimilation, which consequently affects one-carbon metabolism. The involvement of cobalamin, choline, folate, and pyridoxine in the feline one-carbon metabolism is shown in Figure 1.

Cobalamin, a water soluble vitamin that is synthetized solely by anaerobic microorganisms, is a known cofactor for two enzyme systems involved in methionine metabolism. Adenosylcobalamin-dependent methylmalonyl CoA mutase participates in the metabolism of propionate, a metabolite of methionine catabolism, and methylcobalamin-dependent methionine synthetase catalyzes the removal of the methyl group from 5-CH_3_-THF and its transfer to homocysteine, producing methionine and tetrahydrofolate (THF). Cobalamin deficiency in cats has been shown to affect one-carbon metabolism, leading to increased levels of methylmalonic acid and methionine and decreased levels of cystathionine and cysteine, without affecting homocysteine concentrations [76,77]. Cobalamin deficiency can also lead to functional deficiency of folate, further affecting one-carbon metabolism.

Folate is also a water soluble vitamin. Folate coenzymes act as acceptors or donors of one-carbon units in a variety of reactions involved in nucleotide biosynthesis and methyl metabolism. A major cytosolic cycle of one-carbon incorporation involves the reduction of 5,10-methylene-tetrahydrofolate (5,10-CH_2_-THF) to 5-CH_3_-THF, followed by the cobalamin-dependent transfer of the methyl group to homocysteine to form methionine and generate THF. Long-term folate deficiency in cats is associated with weight loss, anemia and leucopenia [78]. Folate deficiency may also affect methionine metabolism, leading to elevated plasma concentrations of homocysteine [79]. Chronic mild hyperhomocysteinemia is recognised as a risk factor for vascular disease in humans [80,81,82], which could not be concluded in cats with cardiomyopathy and arterial thromboembolism [83].

Also choline is linked to lipid and folate-dependent one-carbon metabolism. Choline is not a true vitamin in the classical sense, because many animals are able to synthetize choline in the liver by methylation of ethanolamine. Yet, as the synthesis is inadequate under some conditions and small amounts of choline in the diet can prevent certain pathological conditions, it has been traditional to include choline with the B-vitamins [84]. The main tissue involved in choline metabolism is the liver, where choline has two main roles. As a methyl donor, choline provides active methyl groups for methylation reactions, which includes the formation of methionine from homocysteine involving the choline metabolite, betaine. A second functional role of choline is the biosynthesis of phosphatidylcholine, a structural element of membranes and a required component of VLDL. The mobilization of TAG from liver and its delivery to tissues is accomplished mainly by VLDL. As in many other species, suboptimal concentrations of dietary choline are associated with a diminished capacity of the liver to synthesize phosphatidylcholine resulting in accumulation of lipids in the feline liver [85,86,87].

### 2.3. Essential Fatty Acid Requirement

Most mammalian species readily convert linoleic acid (18:2*n*6) to arachidonic acid (20:4*n*6) by subsequent desaturation and elongation steps [88]. In contrast, early evidence in cats showed a limited capacity to synthetize arachidonic acid from linoleic acid and probably eicosapentaenoic and docosahexaenoic acid from α-linolenic acid (18:3*n*3). This limited synthetic capacity was attributed to lack of ∆6 and ∆5 desaturases in the feline liver [89,90,91]. Cats fed a purified diet containing vegetable oils, which provided essential fatty acids (EFA) only as linoleic acid or as a mixture of linoleic and α-linolenic acid, developed signs of EFA deficiency, and plasma lipids had extremely low levels of arachidonic acid [89]. The lack of desaturases and the essentiality of animal fat as source of arachidonic acid were, however, questioned. The presence of ∆5 desaturase activity was suggested as changes in the fatty acid profile of erythrocyte phospholipids were noted when cats were fed purified γ-linolenic acid (18:3*n*6) [88]. Furthermore, more recent studies using gas chromatography/mass spectrometry (GC/MS) and stable isotope techniques have provided support for the presence of a detectible amount of both ∆6 and ∆5 desaturase products in the feline liver; yet, the activity does not appear to be adequate for maintaining tissue stores of long-chain poly-unsaturated fatty acids (PUFA) [92]. Recently, the presence of an active ∆5 desaturase was also supported by Trevizan *et al.*, showing that γ-linolenic acid provides substrate for arachidonic acid synthesis via by-passing the ∆6 desaturase step [93]. In addition, the ∆6 desaturase enzyme does not appear to be inducible at higher dietary linoleic acid [93].

## 3. Implications for Feline Health—Feline Hepatic Lipidosis

Since its first description [94], FHL has emerged as the most common form of liver disease diagnosed in domestic cats in North America. A 10-year retrospective study demonstrated that FHL accounted for 50% of all feline liver biopsies [95]. In 2008, based on data from primary veterinary healthcare practices across the United States, the prevalence of this syndrome was 0.16% [12].

**Figure 2:**
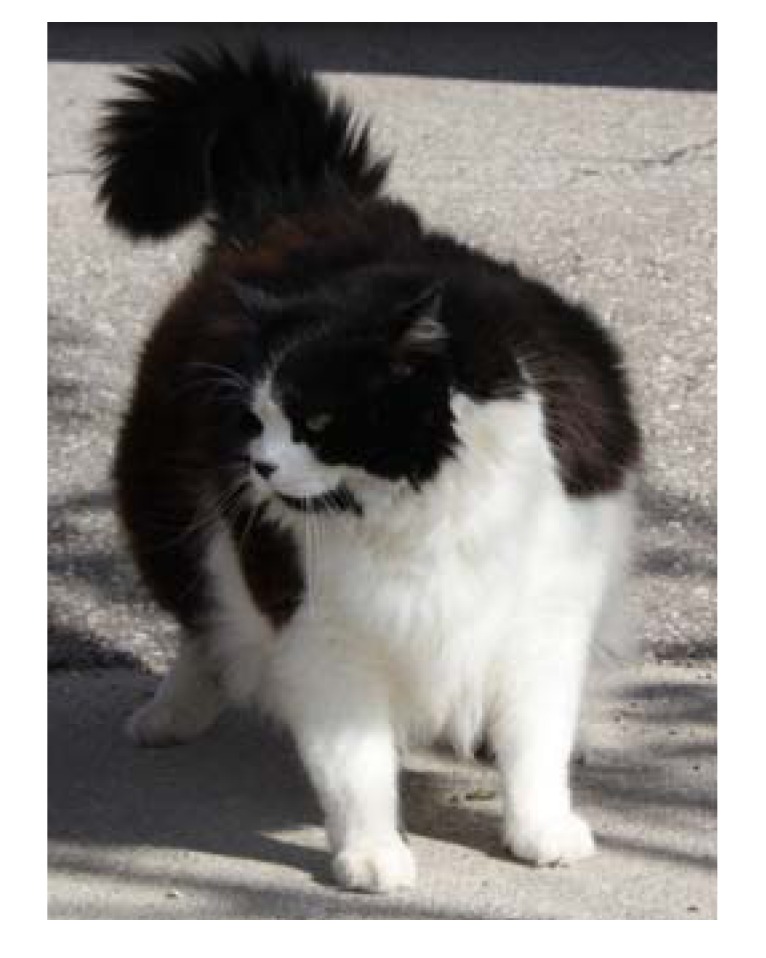
Obesity predisposes cats to hepatic lipidosis.

**Figure 3:**
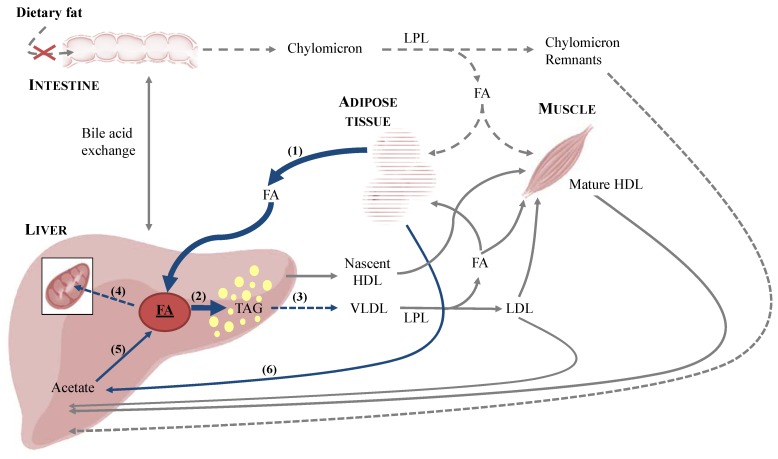
Lipid metabolism in fasting cats. Fasting in cats promotes lipolysis in adipose tissue and transportation of free fatty acids to the liver (**1**). In the liver, fatty acids are reconstituted in triacylglycerol (TAG) (**2**), which are secreted from the liver in very-low-density lipoproteins (**3**) or are transported over the mitochondrial membrane by l-carnitine to enter β-oxidation (**4**). The hepatic load of fatty acids is also increased by *de novo* synthesis of fatty acids, most probably by use of acetate a carbon source (**5**), resulting from ketogenesis (**6**). An imbalance between these different aspects of the feline lipid metabolism leads to accumulation of lipids in the liver, represented as yellow fat droplets. HDL: high-density lipoproteins; FA: fatty acids; LDL: low-density lipoproteins; LPL: lipoprotein lipase; NEFA: non-esterified fatty acids; TAG: triacylglycerols; VLDL: very-low-density lipoproteins.

**Figure 4:**
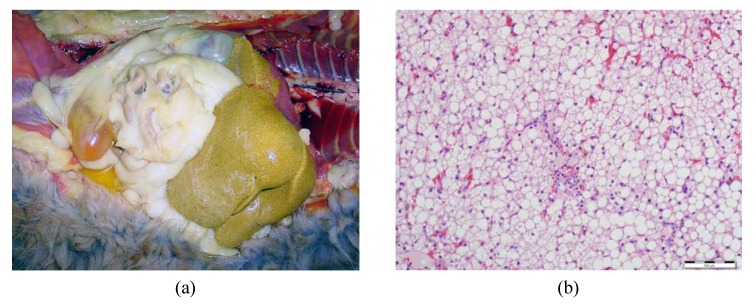
The liver of an obese cat with hepatic lipidosis. (**a**) *In situ* liver of an obese cat with hepatic lipidosis; note the yellow discoloration and hepatomegaly. (**b**) Liver tissue of an obese cat with hepatic lipidosis, showing diffuse cytoplasmic vacuolization of the hepatocytes representing fat accumulation (H & E staining, original magnification: 20×).

Although the precise pathogenesis of FHL remains a mystery, most researchers believe that multiple factors associated with the cat’s unique pathways of protein and lipid metabolism are involved. Proposed pathophysiologic mechanisms may include metabolic changes associated with starvation, insulin resistance, obesity (Figure 2), protein and amino acid deficiency, l-carnitine deficiency, reduction of antioxidant availability, B-vitamin deficiency and essential fatty acid deficiency [12,13,14]. Overall, as summarised in Figure 3, the disease is characterised by an accumulation of lipids in the liver, due to an imbalance between peripheral fat stores mobilised to the liver, *de novo* synthesis of fatty acids, hepatic use of fatty acids for energy and redistribution of hepatic TAG, leading to a fatty liver and an impairment of liver function [96,97]. Figure 4 shows images of the liver of an obese cat with FHL; note the yellow discoloration and hepatomegaly in the *in situ* picture (Figure 4a) and the cytoplasmic vacuolization of the hepatocytes representing fat accumulation on the histological slide (Figure 4b).

### 3.1. Onset of Feline Hepatic Lipidosis

#### 3.1.1. Anorexia and Insulin Resistance

Although many cats develop FHL during periods of anorexia, due to underlying disease (secondary FHL), healthy cats may also develop FHL (primary FHL) due to inadequate intake during forced weight loss, unintentional food deprivation, change to food unacceptable to the cat or stressful conditions, such as sudden change in diet, changes of household or owner, introduction to new people or animals in the house, boarding, *etc*. [11,12]. Biourge *et al*. [98] confirmed that long-term fasting may induce clinical FHL in obese cats. Clinical signs and laboratory results consistent with FHL were observed after five to seven weeks of voluntary fasting, as cats were unwilling to eat an unpalatable purified diet. These clinical signs and laboratory results were associated with a 30% to 35% weight reduction [98]. Histologic examination of liver biopsies revealed that obesity was not associated with accumulation of lipid in the liver, but that fasting resulted in progressive hepatocyte lipid accumulation in all cats beginning at two weeks [98]. In other strict carnivores, such as the European Polecat (*Mustela putorius*) and American Mink (*Neovison vison*), the onset of hepatic lipidosis seems to occur much more quickly. European Polecats, the wild form of domestic ferret, showed hepatic steatosis already after five days of food deprivation [99,100], and the American Mink had fatty livers already after two to three days of fasting [101,102]. Clinical experience indicates, however, that in cats, this clinical syndrome can also develop much more quickly, perhaps related to the percent decrease in caloric intake and degree of obesity [12].

The degree of energy restriction needed to induce FHL was identified to be between 50% and 75%. Armstrong found that lipid did not accumulate in the liver when cats were fed 60% of their calculated maintenance energy requirement for 14 weeks, but did accumulate when fed only 25% [14]. According to Dimski *et al.*, cats fed 50% of the calculated maintenance energy requirement for 29 days did also not develop clinical signs, routine serum biochemical values, lipoprotein electrophoretic patterns and morphologic changes of the liver indicative for FHL [103]. Both studies showed no differences between obese and non-obese cats in terms of response to food restriction. Still, obesity likely predisposes cats to FHL during periods of reduced food intake, because of the quantity of fatty acids that can be rapidly released from peripheral fat stores, pre-existing insulin resistance related to obesity [104] and the significantly greater hepatic TAG content [105].

During a period of fasting, peripheral lipolysis is stimulated by catecholamines, adrenaline and noradrenaline. While in the fed state, lipolysis is inhibited by insulin [106]. Therefore, breakdown of body fat may occur when insulin function is inadequate. Impaired glucose tolerance and insulin response to glucose administration were demonstrated in healthy cats undergoing severe caloric restriction and weight loss, and the cats subsequently developed FHL [107]. When cats were returned to a positive energy balance, glucose tolerance and insulin response normalised, and FHL resolved. Furthermore, Brown *et al.* reported lower serum insulin concentrations and insulin to glucagon ratios in cats with FHL compared to controls [108]. This suggests that once energy restriction occurs, poor insulin function may set up a cycle for continued lipolysis and, ultimately, FHL. Lipolysis and mobilization of fatty acids from adipose tissue induces a dramatic increase of the free fatty acid concentration in the blood. That these fatty acids are taken up by the liver is suggested by the similar composition of fatty acids in the liver and the adipose tissue of cats with FHL [95].

#### 3.1.2. *De Novo* Synthesis of Fatty Acids

Furthermore, *de novo* synthesis of fatty acids could take part in the hepatic load of fatty acids. Rouvinen-Watt *et al*. suggested that in mink, the initial development of steatosis takes place in response to fat mobilisation and subsequent increase in circulating free fatty acid concentrations by day 3 of food deprivation [102]. Yet, the results of this study also show that this is followed by the activation of hepatic *de novo* synthesis of fatty acids, as an increase in the levels of mRNA encoding for acetyl-CoA carboxylase-1 (ACC-1) and fatty acid synthase (FAS) was observed at day 5 and 7 of fasting [102]. It is however important to note that while the liver is the primary site for *de novo* synthesis of fatty acids in humans and rodents [109], in cats, adipose tissue serves this function, followed by liver, mammary glands and muscle [110]. Moreover, while glucose is the precursor in humans [109], in cats, fatty acids are not synthetized at all from glucose by the feline liver, and acetate is the predominant carbon source [110]. It was suggested in mink that acetate could be the substrate for hepatic *de novo* synthesis of fatty acids, as a result from incomplete fatty acid oxidation (*i.e.*, ketogenesis) [102], which is typically enhanced in times of increased free fatty acid intake by the liver [111]. Furthermore, food-deprived cats develop hyperketonaemia more rapidly and to a greater degree than dogs during starvation [112,113]. The hypothesis that some *de novo* synthesis of fatty acids occurs is also supported by the increased hepatic concentrations of palmitate in cats with FHL [95]. Overall, the origin of hepatic TAG in cats with FHL is the mobilization of fatty acids from adipose tissue; yet, hepatic *de novo* synthesis of fatty acids may further exacerbate liver fat accumulation.

Once in the hepatocytes, fatty acids follow one of two main pathways. Fatty acids may enter the mitochondria with the help of l-carnitine, undergo β-oxidation and provide energy. An alternative pathway for fatty acid metabolism is to re-esterify them into TAG, which can be accumulated in liver vacuoles or incorporated into VLDL and secreted in the blood. Several theories involving hepatic fatty acid metabolism have been proposed to explain the pathogenesis of FHL.

### 3.2. Metabolic Aspects of Feline Hepatic Lipidosis

#### 3.2.1. Protein Malnutrition, Arginine and Taurine Deficiency

As mentioned above, adult cats require more dietary protein than omnivorous species, due to a higher endogenous glucose demand, higher basal nitrogen requirement, as well as a need for specific essential amino acids. Unable to adapt urea cycle enzymes, aminotransferases and gluconeogenic enzymes to reduced protein intake, cats possess limited ability to adjust protein metabolic pathways to conserve nitrogen. This means that this species, similar to other carnivores, derives a part of its energy requirement from the breakdown of body proteins. In cats with FHL, increased serum liver enzyme activities, normal total protein, normal to mildly subnormal albumin concentrations and normal to subnormal blood urea nitrogen associated with normal creatinine concentrations were observed [114]. Decreased blood urea nitrogen concentration, which was present in 51% of cats with FHL, may be caused by chronic anorexia or insufficient urea-cycle function [114]. In American mink, the increased plasma levels of urea, ammonia, uric acid, the stable or increased plasma urea:creatine ratios, the elevated plasma liver enzyme activities and decreased protein concentrations in liver and muscle tissue also support the hypothesis of the fasting-induced stimulation of proteolysis [115]. Minks and cats presumably use their body protein as a source of metabolic energy, Krebs cycle intermediates and nitrogen during a negative energy balance [115].

Rapid onset of protein malnutrition in anorectic cats may be an important feature of FHL. First of all, a lack of apolipoprotein B100 may occur with protein malnutrition and was proposed as a reason for the diminished ability of the liver to secrete VLDL, leading to lipid accumulation in the liver [114]. This hypothesis is contradicted by reports of hypertriglyceridemia [11,108,116]. Additionally, increased serum concentrations of VLDL and low-density lipoproteins (LDL) associated with a modified composition of some lipoproteins were reported [11]. Hepatic VLDL secretion is actually increased in cats with FHL, but this may not be sufficient to prevent lipid overload of hepatocytes.

Hastened use coupled with an inability for conservation also necessitates a higher dietary intake of essential amino acids, arginine, taurine, methionine and cysteine, for carnivores compared to other species. In American mink, food deprivation resulted in decreased serum amino acid concentrations [115]. Similar, in FHL, plasma concentrations of alanine, citrulline, arginine, taurine and methionine become markedly reduced (>50% reduction) [117]. While proline, serine, arginine, glycine, alanine and citrulline were permanently decreased in fasted mink, several decreases, namely asparagine, isoleucine, leucine, phenylalanine, taurine and valine, documented at two days of fasting were no longer present after three days without food [115].

As mentioned above, arginine, an important urea cycle substrate, is an essential amino acid for cats. With food deprivation, there is no dietary supply of arginine; its synthesis is inadequate [70,73], and its amount in muscles may be diminished as a result of stimulated proteolysis, which has been demonstrated in American mink [115]. Without available arginine, entrance of ammonia to the urea cycle is limited, the ability to detoxify ammonia to urea is compromised, and hyperammonemia may ensue [73]. Shortage of arginine may also further compromise the liver lipid metabolism, as arginine is required for the synthesis of apolipoprotein E [75], which is known to increase the number of VLDL secreted by the liver of mice [118]. Rapid depletion of arginine may be an outcome of competing requirements overlapping at the time of extensive body fat mobilization; namely, meeting the demands for accelerated VLDL synthesis and eliminating increasing amounts of waste nitrogen resulting from augmented gluconeogenesis. Reduced plasma arginine concentrations have been documented in cats with FHL [117].

Cats are unable to synthetize taurine adequately; yet, they have an obligatory use of this amino acid for bile acid conjugation. Plasma bile acid concentrations in cats with FHL are markedly increased, while plasma taurine concentrations are profoundly low [13]. Taurine-deficiency has been shown to increase total liver lipid content and especially the amount of free fatty acids in the liver in cats, most likely caused by the increased lipolysis in peripheral tissue [67]. According to Cantafora *et al*., the large accumulation of fat in the liver of taurine-deficient cats may be the result of a membrane defect that would result in reduced membrane partitioning within the cell, a reduced conversion of free fatty acids to acyl-CoA, a reduced rate of mitochondrial oxidation or an increased activity of lipolytic enzymes [67]. Yet, liver lipid accumulation was not affected by taurine supplementation in cats undergoing weight gain and subsequent weight loss [105].

#### 3.2.2. Importance of Methionine, SAMe and Carnitine for Proper Lipid Metabolism

Interestingly, also, decreased plasma methionine concentrations were reported in FHL [117], whereas methionine was not altered with food deprivation in American mink [115]. Methionine is essential for methylation reactions and substrate entrance into transsulfuration and aminopropylation pathways through its generation of SAMe.

SAMe is a key methyl donor for phosphatidylcholine synthesis required for the export of VLDL from the liver [119]. However, as mentioned before, hypertriglyceridemia and increased serum VLDL concentrations were observed in cats with FHL, making this hypothesis unlikely.

SAMe is also an essential precursor for l-carnitine, a conditionally essential amine synthetized in the liver. Carnitine is required for transport of long-chain fatty acids into hepatic mitochondria where they undergo β-oxidation. Although high circulating, hepatic and skeletal muscle carnitine concentrations and increased urinary elimination of acyl-carnitine occur in cats with FHL [113,120], it remains unclear whether shifts in dispersal, synthesis and availability are appropriate in magnitude for the metabolic circumstances. If the demand for carnitine exceeds its synthesis, a relative deficiency of carnitine would exist, despite of increased concentrations. In cats fed 25% of their energy requirement, hepatic lipid accumulation was minimal when given supplemental carnitine [121], which implements a much higher carnitine requirement for cats with increased mobilization of fat to the liver and, thus, supports the theory of relative carnitine deficiency in cats with FHL. Furthermore, Blanchard *et al*. demonstrated a protective effect of l-carnitine against fasting as plasma fatty acid concentration rose in fasting cats and during FHL in both control and l-carnitine-supplemented cats, but always to a lesser extent when l-carnitine was administered [113]. Furthermore, according to Center *et al*., dietary l-carnitine supplementation appeared to have a metabolic effect in overweight cats undergoing rapid weight loss that facilitated fatty acid oxidation, as demonstrated by the lower respiratory quotient and the increase in palmitate flux rate with l-carnitine supplementation [122].

Moreover, methionine and cysteine function as major thiol donors necessary for hepatocellular GSH production, which is important for hepatocellular protection from oxidant injury [13]. Low hepatic GSH concentrations in the liver of cats with FHL compared to healthy control cats are consistent with reduction of tissue antioxidant availability [123].

#### 3.2.3. B-Vitamin Deficiency

Cats also seemingly require higher amounts of several B-vitamins and are predisposed to depletion during starvation. Because of their role as a methyl donor in one-carbon metabolism, cobalamin, choline and folate insufficiency may evoke metabolic changes that could play an important role in the pathophysiology of FHL.

As in humans with NAHLD [124], plasma cobalamin concentrations in cats with FHL were lower than those of healthy control cats [12]. Cobalamin is necessary for the synthesis of methionine from homocysteine, an essential reaction when methionine intake is diminished by starvation, as occurs in FHL. Possibly, cobalamin deficiency augments the metabolic changes that promote syndrome onset, especially as limited methionine has an impact on the availability of SAMe and, thereby, secondarily limits the functioning of the transmethylation and transsulfuration pathways [13]. Furthermore, cobalamin deficiency may impair propionate metabolism by decreasing methylmalonyl-CoA activity and may therefore reduce the availability of free carnitine necessary for transport of long-chain fatty acids into mitochondria, where they undergo β-oxidation [13]. The relationship between cobalamin sufficiency, carnitine and hepatic lipidosis has been explored using a rat model of cobalamin deficiency, where carnitine supplementation only partially reversed the metabolic blockade of fatty acid oxidation and hepatic lipid accumulation without restitution of cobalamin [125].

In cats, lipid accumulation in the liver has also been reported with suboptimal concentrations of dietary choline [85,86,87]. Already in 1991, Biourge *et al*. suggested choline supplementation in cats with FHL to ensure it was not limiting for phospholipid synthesis necessary for VLDL production [126]. Moreover, as choline is a major dietary source of methyl donors, and conversion of choline to betaine offers a folate-independent pathway for the remethylation of homocysteine to methionine, the availability of SAMe may also be affected by choline and/or betaine insufficiency. In livestock, betaine, a choline metabolite, has been recognised to prevent liver lipid accumulation by improving VLDL secretion and fatty acid oxidation [127].

No data is available on the role of folate in FHL. Yet, as folate coenzymes are involved in recycling of methionine, folate deficiency may play an important role in the pathophysiology of FHL. In mice, chronic folate insufficiency was shown to lead to steatosis due to increased utilization of betaine for homocysteine remethylation to methionine [128].

At last, in rats fed an obesogenic diet, dietary supplementation with various labile methyl donors, including cobalamin, choline, betaine and folate, reduces fatty liver [129]. As the complex connection between liver function, one-carbon metabolism and energy metabolism is being elucidated, methyl donors will become increasingly important in the treatment and prevention of FHL and hepatic steatosis in various species.

#### 3.2.4. Essential Fatty Acid Deficiency

Development of hepatic steatosis is a well-established manifestation of EFA deficiency in animal models, including the cat [130], and is most probably a combined result of decreased fatty acid oxidation, enhanced *de novo* lipogenesis and altered hepatic VLDL secretion [131]. An abundance of poly-unsaturated fatty acids (PUFA) regulates numerous genes targeted by peroxisomal proliferator activated receptor-α (PPARα), especially inducers of hepatic fatty acid oxidation [111,132]. PUFA are also physiological suppressors of fatty acid synthesis directly through down-regulation of sterol regulatory element binding protein-1 (SREBP1c) [111,133,134] and indirectly, because of inactivation of liver X receptors (LXR) [111]. Furthermore, EFA deficiency does not quantitatively affect hepatic VLDL-TAG secretion, but increases VLDL particle size [131].

Changes in tissue fatty acid composition between control and FHL cats have been noted [95]. In particular, the liver lipids reflect the adipose tissue stores, suggesting that the mobilization of adipose stores may serve as the primary source for liver TAG synthesis and accumulation in FHL [95]. Interestingly, the fatty acid composition of hepatic tissue in cats with FHL showed lesser percentages of the long-chain PUFA, especially arachidonic acid, compared to control cats [95]. Due to the limiting nature of the ∆6 desaturase, the EFA status of the feline may be compromised during food deprivation or rapid weight loss. Since the majority of lipids stored in adipose tissue of healthy felines are saturated and monounsaturated fatty acids and the predominant PUFA is linoleic acid, it may be that during food deprivation, the longer chain PUFA (arachidonic and docosahexaenoic acid) are not adequately synthesized, contributing to the pathogenesis of FHL.

Not only inadequate desaturation, but also derangement of the *n*-6/*n*-3 PUFA ratio plays a major role in regulating both fat accumulation and its elimination by the liver [135]. In American mink and European polecats, fatty acid data of various adipose tissue depots and liver tissue clearly showed loss of the *n*-3 PUFA and an increase in the *n*-6/*n*-3 PUFA ratio in response to food deprivation, which could trigger an inflammatory response in the liver tissue and could therefore be a key contributor to the pathophysiology and progression of liver steatosis [99,136]. Hall *et al*. noted lower concentrations of both total *n*-6 and total *n*-3 PUFA in adipose tissue of cats with FHL compared to controls [95]. The *n*-3/*n*-6 PUFA ratio was not statistically assessed in this study; yet, it appears that in cats, the *n*-3/*n*-6 PUFA ratio in liver and adipose tissue is also higher in cats with FHL (liver: 45.6; adipose tissue: 46) compared to control cats (liver: 12; adipose tissue: 14) [95].

## 4. Conclusions

This review demonstrates the importance of understanding the peculiarities of feline metabolism and the perturbed molecular mechanisms that occur with obesity and energy restriction to fully understand the pathophysiology underlying FHL and to develop and implement new strategies to prevent and treat FHL. These strategies should not only aim at maintaining an adequate food intake and achieving safe weight loss in obese cats, but should also focus on dietary supplementation of essential amino acids, EFA, l-carnitine, SAMe and labile methyl donors, such as cobalamin, choline, betaine and folate. Importantly, the complicated link between liver function, one-carbon metabolism and energy metabolism is just beginning to be elucidated and requires further investigation. In light of the similarities between human and feline metabolic diseases, including obesity, diabetes, but also hepatic steatosis, cats are proposed as an excellent model for the study of mammalian metabolic diseases with a specific focus on one-carbon metabolism.
